# An Evaluation of the Effect of the Guide Socket on the Primary Stability of the Miniscrew in a Polyurethane Cortical Bone Model

**DOI:** 10.3390/polym17070962

**Published:** 2025-04-01

**Authors:** Levent Ciğerim, Nazlı Hilal Kahraman

**Affiliations:** Department of Oral and Maxillofacial Surgery, Faculty of Dentistry, Van Yuzuncu Yil University, 65090 Van, Turkey; nazlihilalkahraman@yyu.edu.tr

**Keywords:** polyurethane, primary stability, guide socket, predrilling, miniscrew, insertion torque, removal torque

## Abstract

Only a few studies in the literature have evaluated the effect of pilot drilling to create a guide socket on the primary stability of miniscrews. The aim of this study was to evaluate the effect of creating a guide socket during miniscrew insertion on the primary stability of the miniscrew in an in vitro polyurethane (PU) cortical bone model. PU blocks with a density of 0.96 g/cm^3^ were used for in vitro cortical bone modelling, and 1.8 × 8 mm self-drilling mushroom-head titanium miniscrews were used. A 1 mm diameter surgical fissure drill was used to create the guide sockets in the study groups. A total of 45 miniscrews were included in this study in the no-guide, 2 mm guide, and 4 mm guide groups. The insertion torque values for the miniscrews in the 4 mm guide socket group were significantly lower than in the other groups, and the removal torque values for the miniscrews in the 2 mm guide socket group were significantly higher than in the other groups (*p* < 0.01). This study demonstrated that a 0.96 g/cm^3^ PU block can be used for in vitro cortical bone modelling and that the creation of a 2 mm guide socket increases the primary stability of the 1.8 × 8 mm mushroom-head miniscrew.

## 1. Introduction

Miniscrews are used for a variety of purposes in modern dentistry and are often preferred in the treatment of malocclusions, particularly in orthodontic treatment. Anchorage, defined as the resistance to unwanted tooth movement, is a prerequisite for the treatment of dental and skeletal malocclusions. Controlling the anchorage helps prevent unwanted tooth movement. However, even a small reactive force can cause unwanted movement; to avoid this, absolute anchorage is important [1]. Skeletal anchorage systems such as dental implants, miniplates, and micro- or miniscrews are systems that aim to provide absolute anchorage by preventing the loss of anchorage. Among these systems, miniscrews are often preferred by dental professionals due to their advantages, including the ease of clinical application, long-term stabilisation with immediate loading, relative patient comfort, and cost-effectiveness [2]. Miniscrews are generally mechanically attached to the bone, not osseointegrated, and are commonly used by orthodontists and oral and maxillofacial surgeons [3,4]. Miniscrews have a wide range of applications, particularly in orthodontic treatment. They are used for the correction of deep malocclusions; closure of extraction gaps; correction of inclined occlusal planes; alignment of the midline of teeth; extrusion of impacted teeth; intrusion, distalisation, and mesialisation of teeth; alignment of third molars; and correction of sagittal and transversal malocclusions [5].

Miniscrews are generally available in two types: self-drilling and self-tapping. Both self-tapping and self-drilling screws are effective anchorage devices [6]. Self-drilling miniscrews can be used to correct deep malocclusions, close extraction gaps, correct inclined occlusal planes, align the midline, extrude impacted canines, extrude and erect impacted molars, and self-tapping screws are routinely used to fix maxillofacial fractures and in orthognathic surgery [1,7]. Self-tapping miniscrews have a body with threads evenly spaced along its length and a blunt, tapered tip. A cutting groove is milled into the tip to facilitate removal of bone debris. The body of the self-drilling mini-screw has threads aligned along a rotational axis from the tip to the screw head. The tip has a triangular structure to ensure good placement and simultaneous removal of debris [8]. Self-drilling miniscrews have been reported to have higher insertion and removal torques in various studies [9]. However, it has been reported that self-drilling miniscrews have a tendency to fracture and have a higher bone implant contact surface area [10]. In addition, the time-saving effect of self-drilling systems is less than expected, but self-drilling systems may be advantageous in cases where drilling is technically difficult or even impossible [9]. When the insertion techniques of self-tapping and self-drilling mini-screws were evaluated in terms of stress concentration and deformation at the screw–bone interface, self-tapping screws showed greater deformation than self-drilling screws. It has been reported that self-tapping screws may have a higher incidence of fatigue than self-drilling screws [7].

The advantage of self-drilling miniscrews is that they do not require pilot drilling during the insertion procedure. The miniscrews that are used in orthodontics differ from those that are used in oral and maxillofacial surgery, particularly in the design of their heads. Orthodontic miniscrews allow the appropriate mechanics to be connected to the screw to ensure absolute anchorage. Two types of orthodontic miniscrews are generally available: mushroom-head and bracket-head screws [11,12,13]. In cases where the cortical bone is thicker than 2 mm, pilot drilling may also be required for self-drilling screws, as the applied force will bend the tip of the screw. It has been recommended that if pilot drilling is required, it should be performed by an oral and maxillofacial surgeon. Information on the required depth and width of the guide socket according to the mini-hole that will be used is limited and advisory, but it is generally recommended that they guide socket should be minimal [8].

Polyurethane (PU) is a polymeric material that can have a very wide range of chemical and physical properties, owing to the possibility of modifying its structure or production methods. It can therefore be adapted to different requirements in several fields (coatings, adhesives, thermoplastics) [14]. Due to its mechanical properties, PU is recommended for use in in vitro bone modelling [10,11], and because of its homogeneous and stable physical properties, it is preferred for modelling alveolar bone in in vitro studies to test different implant materials and standardise procedures by eliminating anatomical and structural differences in bone [12,13,14,15].

In general, human bone is not homogeneous. Its physical properties vary greatly by species, age, sex, and type (e.g., femoral, mandibular, cortical and trabecular), and even by the site of sample collection. This complex and heterogeneous nature of human bone, as well as ethical issues, complicates the conduct of clinical trials [16,17,18,19]. Given the difficulties of working with human cadaveric and animal bone, synthetic polyurethane foams have been widely used as alternative materials to bone in various biomechanical tests due to their similar void structure, density and consistent mechanical properties [20]. The literature shows that PU with different densities and properties is preferred as a bone model material in various miniscrew and implant studies where tensile tests, resonance frequency analyses, insertion, and removal torque values were investigated [16,17,18,19,20,21]. In these studies using polyurethane blocks to simulate bone, the effects of the screw’s shape, length, and design, which are among the factors influencing the primary stability of miniscrews, were investigated [22]. There are only a few studies in the literature that have evaluated the effect of pilot drilling to create a guide socket on the primary stability of miniscrews [23,24]. Due to the difficulty of working in cortical bone, we believe that the creation of a guide socket will not only structurally damage a self-drilling miniscrew but also facilitate the initial entry of the miniscrew into the bone, prevent the expansion of the socket (especially the entry portion), and subsequently allow the miniscrew to be properly placed without changing the angle.

The aim of this study was to evaluate the effect of creating a guide socket during miniscrew insertion on the primary stability of the miniscrew by measuring the insertion and extraction torques in an in vitro PU cortical bone model. For this purpose, no guide, 2 mm guide, and 4 mm guide sockets with a width of 1 mm were created and the insertion and removal torques were compared between the groups. This study also aimed to evaluate the use of a single layer PU plate for cortical jaw bone modelling in vitro. The null hypothesis of this study was that the guide socket would have no effect on the stability of the miniscrew.

## 2. Materials and Methods

**Figure 1:**
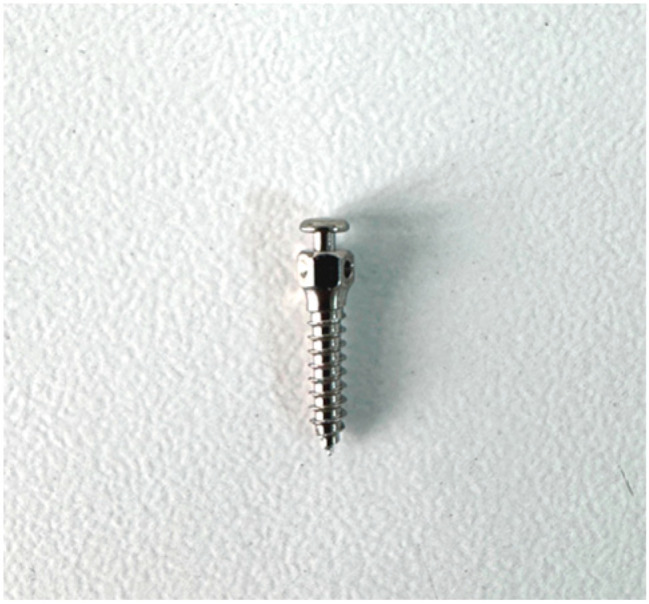
The 1.8 × 8 mm miniscrew used in this study.

**Figure 2:**
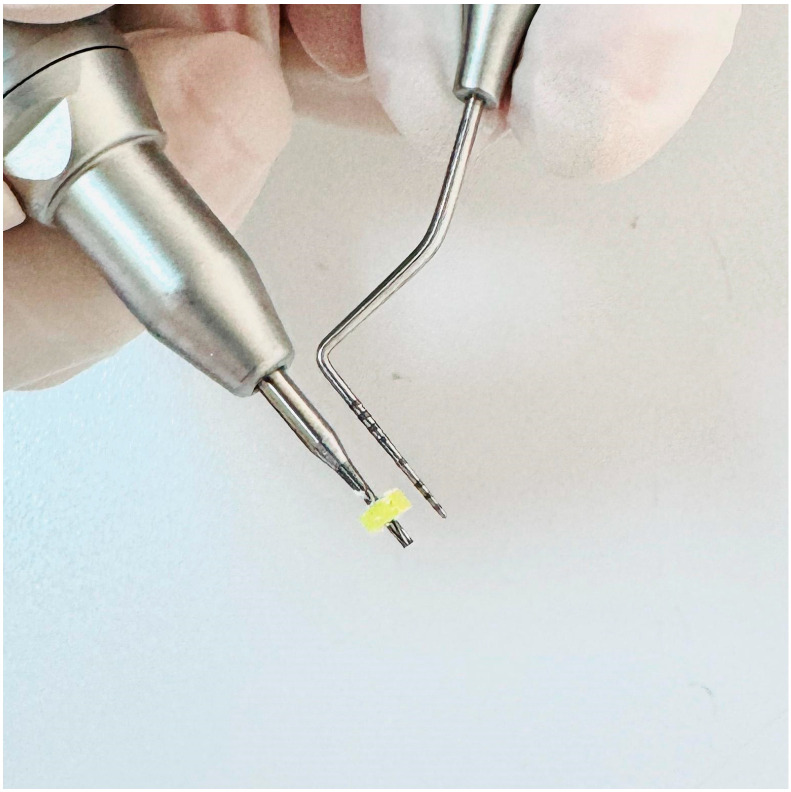
Surgical fissure drill.

This in vitro study was conducted at the Van Yüzüncü Yıl University Faculty of Dentistry, Department of Oral and Maxillofacial Surgery, in December 2024. Polyurethane blocks with 260 × 82 × 20 mm dimensions (Puryap, Construction Chemicals and Machinery Industry Trade Company Limited, Istanbul, Turkey) and a density of 0.96 g/cm^3^ (60 pcf) were used for in vitro cortical bone modelling. Self-drilling mushroom-head titanium miniscrews (Ancor Orthodontics, Ankara, Turkey) measuring 1.8 × 8 mm were used (Figure 1). A 1 mm diameter surgical fissure drill was used to create the guide sockets in the study groups (Figure 2). G*power version 3.1.9.4 was used to calculate the sample size of the study. The effect size was determined from the reference article to be 0.76, and it was calculated that a minimum of 14 subjects per group should be included in the study for an alpha error of 0.01 and 95% power [22]. Accordingly, this study was conducted using a total of 45 miniscrews in 3 groups, with 15 miniscrews in each group. The guide sockets in the study groups were created using a physiodispenser (Straumann Surgical Motor Pro, NSK Nakanishi Inc., Kanuma, Tochigi, Japan) and a 1:1 reduction contra-angle handpiece (NSK Ti-Max X-SG65, Tochigi, Japan) with standard parameters of 1000 revolutions per minute (rpm) and 50 torque under sterile saline. Miniscrews were placed in all groups according to a standardised protocol.

Randomisation was applied to both the creation of the guide sockets in Groups 2 and 3 and the placement of the miniscrews in all groups using an online software programme [25]. In other words, instead of completing one group and moving on to the next, the information on group and procedure order was randomised for each application from the 1st to the 15th application for opening the sockets and placing the miniscrews in each group.

### 2.1. Socket Preparation

The preparation of the guide sockets in Groups 2 and 3, including the 2 and 4 mm guide sockets, was performed using a 1:1 reduction contra-angle handpiece (NSK Ti-Max X-SG65, Tochigi, Japan) with standard parameters of 1000 revolutions per minute (rpm) and 50 torque, with a 1 mm surgical fissure burr. When preparing the sockets, care was taken to ensure that the wall thickness of the sockets and the distance between them were the same to ensure standardisation. The 2 mm and 4 mm guide sockets were prepared by a specialist surgeon who was not involved in the study to ensure blinding of the author surgeons. The surgeon who prepared the sockets checked that they were 1 mm wide and 2 and 4 mm deep, according to the group. Inappropriate sockets were excluded from the study, and new sockets were created to complete the 15 procedures in each group.

### 2.2. Miniscrew Insertion and Removal Procedure

**Figure 3:**
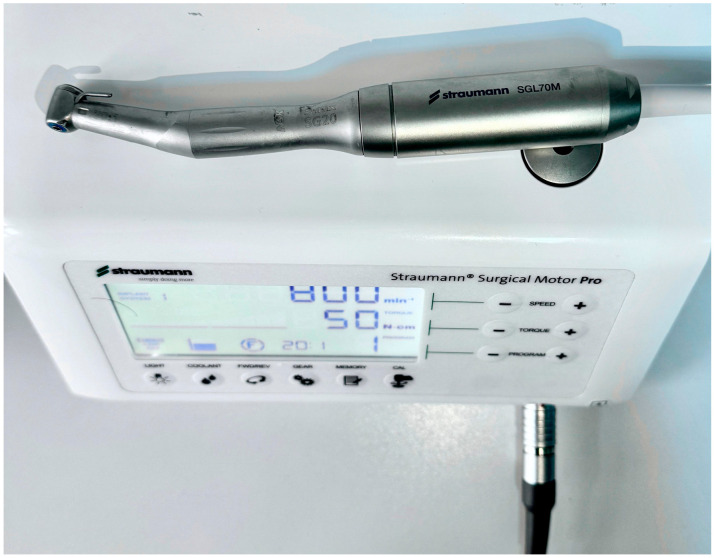
Physiodispenser and implant handpiece.

**Figure 4:**
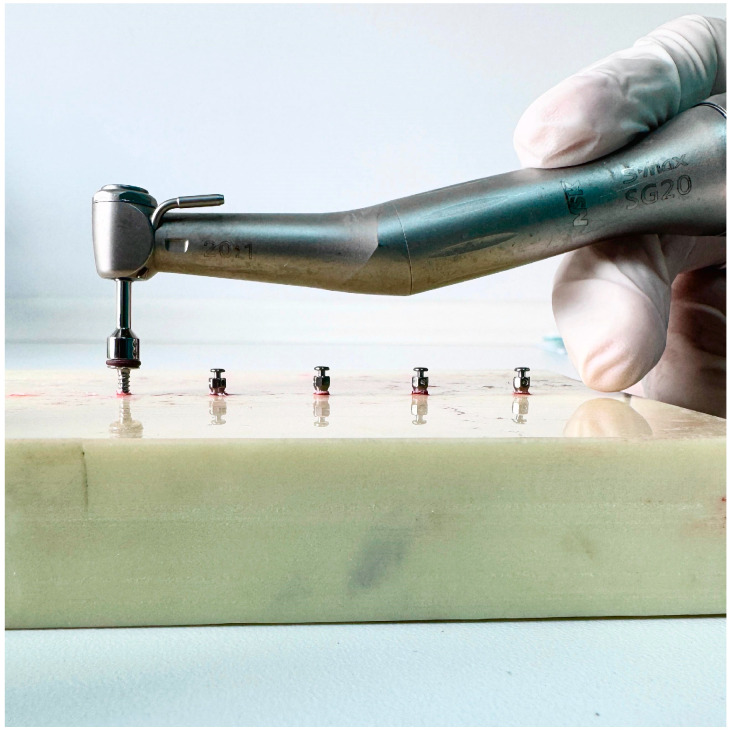
Inserting the miniscrews in Group 1.

**Figure 5:**
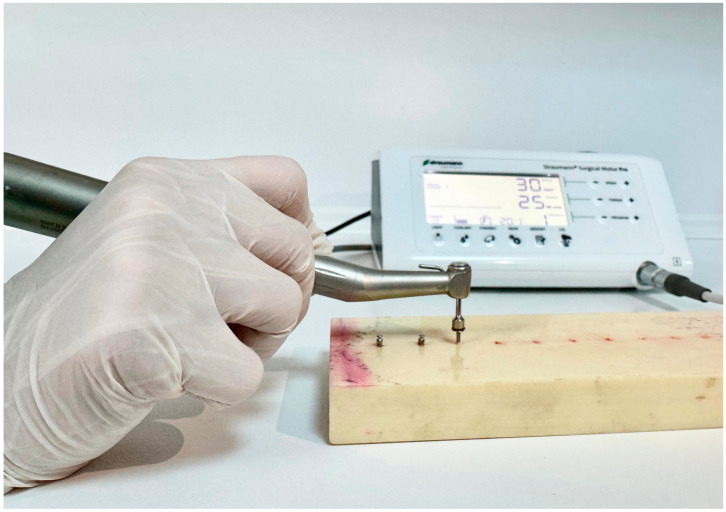
Inserting the miniscrews in Group 2.

**Figure 6:**
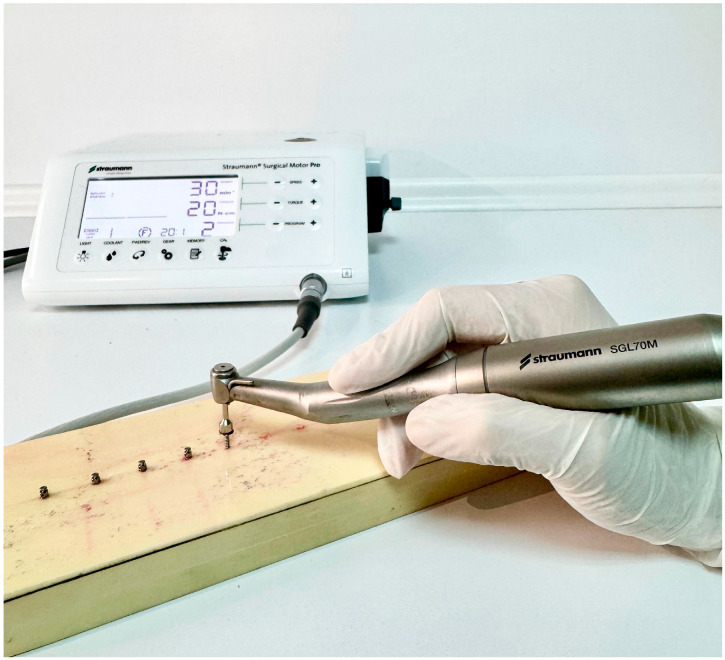
Inserting the miniscrews in Group 3.

In all 3 groups, the miniscrews were inserted by the same author surgeon (NHK). The insertion torques (ITs) and removal torques (RTs) were measured to assess the primary stability of the miniscrews. Miniscrews were placed in all groups using a motorised screwdriver with a physiodispenser (Straumann Surgical Motor Pro, NSK Nakanishi Inc., Kanuma, Tochigi, Japan) and a 20:1 implant handpiece (NSK S-Max SG20, Tochigi, Japan) at 30 rpm (Figure 3). When inserting the miniscrews, the physiodispenser was initially set to a torque value of 5. If the miniscrews did not rotate before being fully inserted, the value was increased by 5 torques (Figure 4, Figure 5 and Figure 6). The last torque value at which the miniscrew was fully inserted in the neck region was recorded as the insertion torque value. The physiodispenser was then operated in reverse mode without removing the miniscrews from the implant handpiece, and the same protocol as for insertion was followed. The lowest torque value that rotated the implant and removed it from the socket was taken as the removal torque. During insertion and removal, the miniscrews were placed perpendicular to the blocks, and no lateral force was applied. The surgeon performed the procedures with their wrists resting on the model to ensure standardisation in the preparation of the guide sockets and insertion and removal of the miniscrews.

### 2.3. Study Groups

The miniscrews included in this study were divided into 3 groups. Group 1 (control group) did not include a guide socket. Self-drilling miniscrews were inserted into the polyurethane block according to the protocol. In Group 2, a 1 mm wide guide socket was inserted. In these sockets, miniscrews were inserted into the polyurethane block according to the established protocol. In Group 3, a 1 mm wide and 4 mm guide socket was created. In these sockets, miniscrews were inserted into the polyurethane block according to the established protocol.

The polyurethane blocks that were used in all 3 groups had a density of 0.96 g/cm^3^. The blocks were manufactured at the same time under the same humidity and temperature conditions. The socket preparation and miniscrew insertion were performed by surgeons to mimic the clinical environment.

### 2.4. Statistical Analysis

SPSS (Statistical Package for the Social Sciences), version 27, was used for statistical analyses in the evaluation of the study results. In the evaluation of the study data, quantitative variables were presented using mean, standard deviation, median, minimum, and maximum values, and qualitative variables were presented using descriptive statistical methods such as frequencies and percentages. The Shapiro–Wilk test and box plots were used to assess the suitability of the data for normal distribution. The Kruskal–Wallis test was used to compare variables that did not have a normal distribution in three or more groups, and the Dunn test was used to determine the group responsible for the difference. Results were evaluated with a 95% confidence interval and significance at the *p* < 0.05 level.

## 3. Results

A total of 45 miniscrews were included in this study in the no guide, 2 mm guide and 4 mm guide groups. The total ITs of the miniscrews were 21.22 ± 4.28 N-cm, and the total RTs were 18.00 ± 2.90 N-cm (Table 1).

**Figure 7:**
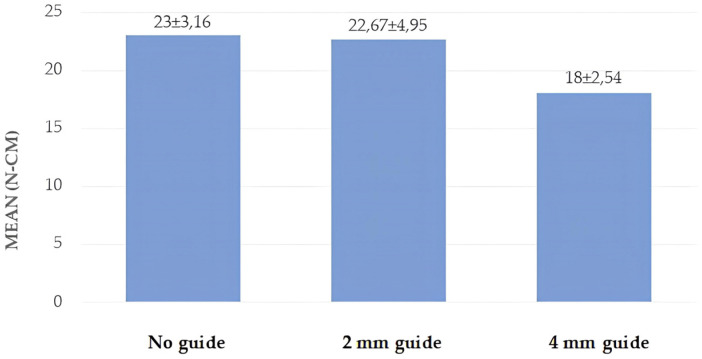
Distribution of insertion torque values by group.

A statistically significant difference was found between the IT measurements of the miniscrews according to group (*p* = 0.001; *p* < 0.01). The IT values of the miniscrews in Group 3 were significantly lower than those in Groups 1 and 2 (*p* = 0.001; *p* = 0.009; *p* < 0.01) (Table 2, Figure 7).

**Figure 8:**
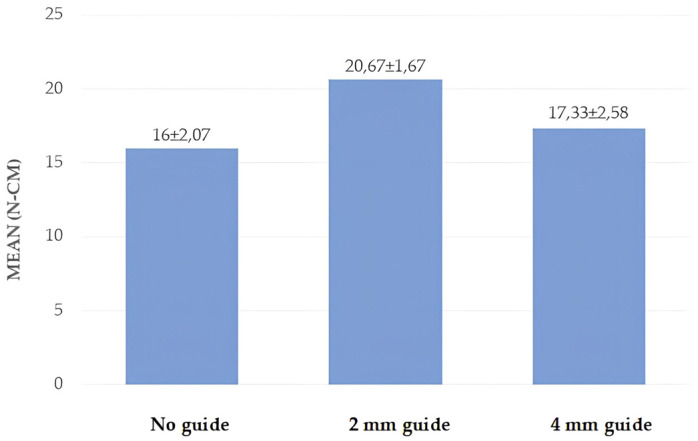
Distribution of removal torque values by group.

A statistically significant difference was found between the RT measurements of the miniscrews according to group (*p* = 0.001; *p* < 0.01). The RT values of the miniscrews in group 2 were significantly higher than those in groups 1 and 3 (*p* = 0.001; *p* = 0.006; *p* < 0.01) (Table 2, Figure 8).

## 4. Discussion

This study evaluated the effect of guide sockets, created on a polyurethane cortical bone model, on the primary stability of a mushroom-head orthodontic miniscrew. According to our results, the creation of guide sockets did affect the primary stability of the miniscrew, and therefore, the null hypothesis was rejected.

Miniscrews are used as temporary anchorage devices because of their immediate loading, ease of insertion and removal, and relatively low cost. The small width of miniscrews allows them to be inserted atraumatically into the alveolar bone between the roots of the teeth. Despite the advantages of self-drilling miniscrews, as all surgical procedures have complications, the use of self-drilling miniscrews has been associated with inadequate anchorage, miniscrew migration, and failure [26]. Mechanical evaluation of the stability of implanted screws, including miniscrews, is usually based on the insertion and removal torque [27]. The stability of the miniscrews is influenced by several factors, such as the insertion technique, insertion angle, length, applied loads, bone density, and cortical bone thickness. Among these factors, the thickness and density of the alveolar bone in which the miniscrews are placed is one of the most important [21,28]. Baumgaertel et al. reported that the insertion torque increased with increasing cortical bone thicknesses [29]. High stability of the miniscrew is desirable for anchorage, but it has been reported that screw-related complications increase with increasing insertion torques during miniscrew placement. Self-drilling miniscrews may be easier to place in areas of thin cortical bone, and studies have also reported that miniscrew fractures may occur in cases where the cortical bone thickness increases [22,28]. These results show that care must be taken when working on thick cortical bone. For this reason, in this study, we used a single layer 0.96 g/cm^3^ polyurethane block to simulate thick cortical bone, which we believe simulates alveolar cortical bone in the clinical environment. In this study, we used a single layer (single density) model for in vitro modelling of the cortical bone of the jaw. Two-layer models were commonly used for corticocancellous modelling [30,31]. As the study investigated the effect of the depth of the guide slot on the primary stability of the miniscrew, it is not possible to achieve standardisation between groups with two-layer models, and as this two-layer structure will directly affect the results, the effect of the guide slot on primary stability will be masked. In other words, in one group the guide slot was only opened in cortical bone of a single density, whereas in the other group the guide slot was opened in cortical bone of two different densities or cortical cancellous bone, so it is clear that this will affect the results. We used a single layer model to avoid the effect of the model material on the results.

To simulate thick cortical bone and evaluate the fracture resistance of miniscrews, some studies have used acrylic models. For example, in Smith’s study, self-drilling screws of similar diameters from six different brands were placed in an acrylic model, with the fracture torques ranging from 25.8 to 72.07 [28]. In another study, Wilmes et al. (2011) placed several different types of miniscrews in acrylic blocks and showed that the miniscrews fractured when using torques ranging from 10.8 N-cm to 64 N-cm depending on the type of miniscrew [32]. In a study using a femoral bone model, Pithon et al. found the fracture torques of various miniscrews to range from 49.6 to 99.15 N-cm [33]. These studies were concerned with the high torque values associated with miniscrew fracture in high-density models simulating thick cortical bone, and given their results, the fracture resistance of miniscrews may vary depending on the model material and miniscrew type [28,32,33,34]. No studies in the literature have evaluated miniscrew fracture in polyurethane blocks. In this study, no screw fracture was observed during the insertion and removal of the miniscrews. It may not be appropriate to compare the results of studies using acrylic and animal models with our results, which were obtained using a polyurethane block. Based on the literature, polyurethane is an accepted material for alveolar bone simulation under in vitro conditions and is similar to bone in its mechanical and physical properties [35,36]. In this study, we chose a polyurethane block for cortical bone modelling in accordance with the literature 

Few studies in the literature investigate the effect of a guide socket on the primary stability of a miniscrew in polyurethane models. Among these, Cho et al. investigated the effect of different guide socket formation methods on miniscrew stability. In their study, they used a two-layer polyurethane block with a density of 0.64 g/cm^3^ and formed two groups according to the thickness of the outer layer. One group’s guide sockets had a 2 mm thick outer layer with a density of 1.632 g/cm^3^, while the other group’s sockets had a 4 mm thick outer layer with a density of 1.632 g/cm^3^. The authors created 5 mm guide sockets with hand and power drills, and the hand drill that they used was 1.6 mm wide. They found that there was no difference in the insertion torques between the hand and power drills, and that the removal torque was higher in the 4 mm group [23]. Similarly, Phusantisampan et al. investigated the effect of a guide socket on miniscrew stability, and 5 mm guide sockets were created using pilot drills of 1.1, 1.2, 1.3, 1.4, and 1.5 mm diameters. The polyurethane block used was a two-layer block, whose inner part had a density of 0.32 g/cm^3^, while the outer part was 2 mm thick and had a density of 0.64 g/cm^3^. According to their results, the insertion torque values of the groups with guide sockets were lower than those of the control group without guide sockets. In their study, they also evaluated the pull-out strength—another method of evaluating stability—and showed that the pull-out strength of the miniscrews that were placed in the sockets formed with small-diameter pilot drills was higher than that of those with larger diameters [24]. In another study using two-layer polyurethane blocks, Heo et al. used polyurethane blocks with a 3 mm thick outer part and a density of 0.8 g/cm^3^, as well as an inner part with a density of 0.48 g/cm^3^. In their study, they created guide sockets with depths of 1.5 and 4 mm and an angle of 60 degrees. They compared the stability of the miniscrew in guide sockets of different depths with the control group without guide sockets and found that there was a decrease in the insertion torque in both groups compared with the control group; however, the decrease was greater in the 4 mm group [37]. In the study by Cho and Baek [38], using the same polyurethane model and 1mm pilot drill as in the study by Heo et al. [37], the insertion torques of guide sockets that were prepared at depths of 1.5 and 3 mm with a 90-degree angle were compared with the control group. The results showed that the insertion torque value in the control group, i.e., the group without a guide socket, was higher than in the other groups, in which guide sockets were used. The authors mainly attributed this decrease to the drilling of the cortical part in the blocks with guide sockets and the thickness of the supported cortical part [38]. Although all these studies used two-layer polyurethane blocks, the outer and inner layer thicknesses and densities of the blocks were different. Despite these differences, it can be seen that cortical bone was modelled in all three studies, and that as the density of the outer layer, which is the dense part in the models used, increases, so does the stability of the miniscrew. The insertion torques in the control groups in these studies ranged from 11.58 to 29.75 N-cm. Unlike the above studies, our study used a single-layer polyurethane block with a density of 0.96 g/cm^3^. In this study, the insertion torque values obtained for the control group were found to be 23 N-cm, in agreement with the literature, which supports the use of the blocks that we used when modelling cortical bone. In the present study, using screws of the same diameter as in the previous studies, it was observed that—unlike in the previous studies—the formation of the guide socket did not reduce the stability of the miniscrew; on the contrary, the guide socket that was formed at a depth of 2 mm did not affect the insertion torque value of the miniscrew but increased the removal torque value. When the depth of the guide socket was increased to 4 mm, it was observed that the insertion torque value decreased, but the removal torque value did not change. We believe that this difference in our study was due to the different density and thickness of the polyurethane model used as well as the different depths and diameters of the prepared guide sockets. Cho and Baek and Heo et al. showed that the insertion torque values decreased as the guide socket diameter remained the same and the depth increased. Similarly to these studies, in the present study, the miniscrew insertion torque value of the 4 mm depth guide socket was lower than that of the 2 mm depth guide socket.

The use of artificial bone in mechanical studies may lead to different results to those obtained with human bone. The main reason for this is the inhomogeneous bone density at the insertion site in human bone. To control for bone density as a variable, it is advantageous to use experimental artificial bone with homogeneous density values and a linear direction during insertion [29]. Therefore, a bone model with a density of 0.96 g/cm^3^ which is similar to the density of the jaw bone, was used in this study. Although self-drilling screws are used to increase the stability of miniscrews, predrilling can be considered in all areas of high bone density. The diameter and depth of predrilling should be appropriate for the bone quality and the area in which the miniscrew will be placed [39]. Self-drilling miniscrews may be subject to dislocation under orthodontic loading. Although the behaviour of self-drilling miniscrews under orthodontic loading is not clinically clear, a miniscrew that is placed in the interdental region may be displaced under loading, and a serious problem may occur if it contacts the adjacent tooth roots [26]. For this reason, we believe that increasing the stability of the miniscrew may be beneficial. In this study, the effect of guide socket formation on the primary stability of a miniscrew was analysed. The 2 mm and 4 mm guide socket study groups were compared with the no-guide-socket group. Higher removal torques were achieved in the 2 mm guide socket group compared with the control group and the 4 mm guide socket group. Lower insertion torques were achieved in the 4 mm guide socket group compared with the other two groups. According to these results, displacements at the initial bone entry may lead to wider socket formation, which may reduce stability. This demonstrates that with a guided socket, we can achieve a more stable socket and therefore increase the primary stability. The 2 mm guide socket group had maximum removal torques and we believe its clinical importance is that it can be used to increase the primary stability of the miniscrew in cases where absolute anchorage is required or in cases where primary stability is thought to be low. Secondly, in cases where there is sufficient primary stability in the cortical bone but the miniscrew is difficult to place, we believe it will reduce the complications associated with the miniscrew by allowing less damage to the miniscrew without loss of primary stability. Increasing the depth of the socket may decrease the contact between the screw and bone and therefore decrease the stability. The length of the miniscrew used is 8 mm and we believe that the reason why the 2 mm guide has a higher removal torque value than the 4 mm, even though the guide sockets prevent expansion of the sockets and changes in placement angle, is because the surface of the miniscrew in the 2 mm guide (6 mm of undrilled bone present) provides more bone (model) contact than in the 4 mm guide (4 mm of undrilled bone present). These results show that mushroom head miniscrews can be used in dentistry with an appropriate guide socket when skeletal anchorage is required. In cases where the cortical bone is dense and thick, we observed that a guide socket of appropriate depth and diameter can increase or decrease the stability of the miniscrew. This means that clinicians can use guide socket in cases where they want to achieve reduced primary stability to protect against miniscrew-related complications and/or in cases where they want to achieve increased primary stability to achieve better skeletal anchorage. Thus, the use of the appropriate guide socket allows the clinician to manage the unfavourable and challenging characteristics of the cortical bone.

The limitations of this study were as follows: although the guide sockets in this study were created by a surgeon who was not part of the study, there may have been minimal variation in the sockets due to application. The miniscrews were inserted by a surgeon who was conducting the study, and there may have been minimal differences in the balance of forces applied during insertion. The study used 2 mm and 4 mm sockets, and different widths and depths of the sockets may have produced different results. At the same time, the choice of different densities of the polyurethane block used in this study may also have caused differences in our results.

## 5. Conclusions

In this study, the creation of a guide socket at a depth of 2 mm was found to increase the primary stability of the 1.8 × 8 mm mushroom head miniscrew. As the depth increased to 4 mm, the stabilising effect of the guide socket decreased. These results demonstrate that a guide socket can be used to both increase and decrease the primary stability required by the clinician when placing a miniscrew in cortical bone. This study also showed that a single layer polyurethane plate with a density of 0.96 g/cm^3^ can be used for in vitro cortical jaw bone modelling. Further studies are needed to investigate the effect of guide sockets with different depths and diameters on models with different densities.

## Figures and Tables

**Table 1:** Descriptive characteristics of groups.

		n (%)
Group	1 (no guide socket)	15 (33.3)
	2 (2 mm guide socket)	15 (33.3)
	3 (4 mm guide socket)	15 (33.3)
IT N-cm	mean ± sd	21.22 ± 4.28
	minimum–maximum	15–35
RT N-cm	mean ± sd	18.00 ± 2.90
	minimum–maximum	15–25

IT: insertion torque, RT: removal torque.

**Table 2:** Comparison of insertion and removal torque values according to group.

		Group 1 (n = 15)	Group 2 (n = 15)	Group 3 (n = 15)	^a^ *p*
IT N-cm	mean ± sd	23.00 ± 3.16	22.67 ± 4.95	18.00 ± 2.54	0.001 *
	minimum–maximum	20–30	20–35	15–20	
RT N-cm	mean ± sd	16.00 ± 2.07	20.67 ± 1.76	17.33 ± 2.58	0.001 *
	minimum–maximum	15–20	20–25	15–20	

^a^ Kruskal–Wallis and Dunn–Bonferroni tests; * *p* < 0.01. IT: insertion torque, RT: removal torque.

## Data Availability

The data presented in this study are available on request from the corresponding author due to legal reasons.
